# Improvement of activity and stability of chloroperoxidase by chemical modification

**DOI:** 10.1186/1472-6750-7-23

**Published:** 2007-05-18

**Authors:** Jian-Zhong Liu, Min Wang

**Affiliations:** 1 Key Laboratory of Gene Engineering of Ministry of Education and Biotechnology Research Center, State Key Laboratory of Biocontrol, Zhongshan University, Guangzhou 510275, PR China

## Abstract

**Background:**

Enzymes show relative instability in solvents or at elevated temperature and lower activity in organic solvent than in water. These limit the industrial applications of enzymes.

**Results:**

In order to improve the activity and stability of chloroperoxidase, chloroperoxidase was modified by citraconic anhydride, maleic anhydride or phthalic anhydride. The catalytic activities, thermostabilities and organic solvent tolerances of native and modified enzymes were compared. In aqueous buffer, modified chloroperoxidases showed similar *K*_m _values and greater catalytic efficiencies *k*_cat_/*K*_m _for both sulfoxidation and oxidation of phenol compared to native chloroperoxidase. Of these modified chloroperoxidases, citraconic anhydride-modified chloroperoxidase showed the greatest catalytic efficiency in aqueous buffer. These modifications of chloroperoxidase increased their catalytic efficiencies for sulfoxidation by 12%~26% and catalytic efficiencies for phenol oxidation by 7%~53% in aqueous buffer. However, in organic solvent (DMF), modified chloroperoxidases had lower *K*_m _values and higher catalytic efficiencies *k*_cat_/*K*_m _than native chloroperoxidase. These modifications also improved their thermostabilities by 1~2-fold and solvent tolerances of DMF. CD studies show that these modifications did not change the secondary structure of chloroperoxidase. Fluorescence spectra proved that these modifications changed the environment of tryptophan.

**Conclusion:**

Chemical modification of epsilon-amino groups of lysine residues of chloroperoxidase using citraconic anhydride, maleic anhydride or phthalic anhydride is a simple and powerful method to enhance catalytic properties of enzyme. The improvements of the activity and stability of chloroperoxidase are related to side chain reorientations of aromatics upon both modifications.

## Background

Chloroperoxidase (CPO; EC 1.11.1.10) is a heavily glycocylated monomeric hemoprotein secreted from the filamentous fungus *Caldariomyces fumago*, with a sugar content of 18% of its molecular mass of 42 kDa [1,2]. Comparison of the X-ray crystallographic structure of CPO [3] with other known peroxidases, namely cytochrome C peroxidase [4], lignin peroxidase [5], peroxidases from *Coprinus cinereus *[6], peanut peroxidase [7], and horseradish peroxidase isoenzyme C [8], reveals dramatic structural differences between CPO and these traditional heme peroxidases. For example, the proximal axial ligand of the heme iron in CPO is a cysteine (Cys 29) sulfur atom in common with the cytochromes P450 (CYP450s) rather than a histidine nitrogen atom commonly found in the traditional heme peroxidases. Moreover, Glu 183 is postulated to function on distal side of the heme prosthetic group as an acid-base catalyst in facilitating the reaction between the peroxidase and hydrogen peroxide with the formation of Compound I. In contrast, most other heme peroxidases use a histidine to fulfil the same function [3]. These structural features shared with both heme peroxidases and cytochromes P450 make CPO the most versatile of the known heme enzymes. In addition to catalyzing chlorination, bromination, and iodination reactions [9], CPO also catalyses other reactions characteristic of heme peroxidases (dehydrogenation), catalases (H_2_O_2 _dismutation) and cytochromes P450 (monooxygenation) [1,10]. Most importantly, CPO is especially adept in catalyzing the stereoselective epoxidation of alkenes [11], benzylic hydroxylation [12], propargylic oxidation of 2-alkyenes to chiral alcohol [13] and oxidation of organic sulfides to chiral sulfoxides [14,15]. The stereoselective abilities of CPO in chiral catalysis suggest that native CPO and/or engineered CPO mutants has the potential to become important industrial catalysts [16]. However, CPO is readily inactivated above 50°C [17], limiting its use in many fields.

In general, enzymes show relative instability in solvents or at elevated temperature and lower activity in organic solvent than in water. These limit the industrial application of enzymes. Chemical modification has now re-emerged as a powerful complementary approach to site-directed mutagenesis and directed evolution for improving activity and stability of enzymes [18,19]. Many research groups demonstrated that chemical modification of enzyme could enhance its activity or stability by different dicarboxylic anhydrides, such as phthalic anhydride (PA), maleic anhydride (MA), citraconic anhydride (CA) and succinic anhydride [20-28]. These anhydrides react specifically with the ε-amino group of lysine residues and change its charges from positive to negative. Our previous papers reported that horseradish peroxidase was chemically modified to enhance its activity and stability by phthalic anhydride, maleic anhydride or citraconic anhydride [20-24]. Hernandez et al. reported that the modified CPO by reductive alkylation, amidation and cross-linking showed higher stability than the native enzyme in 60% *tert*-butanol [29]. It has also been demonstrated that addition of polyethylene glycol 200, even without modification, enhanced the activity and stability of chloroperoxidase [30].

In this study, CPO was chemically modified by phthalic anhydride, maleic anhydride or citraconic anhydride. The changes of activity and stability after modification were investigated. The aim was to understand how these modifications affect its activity, stability and structure.

## Results and discussion

### Activity and kinetics

Modification of lysine residues (figure 5) was carried out by following the procedure described in the Methods section. In order to optimize the ratio of the modified reagent and the enzyme, effects of the concentration of the modified reagents on activity at a fixed enzyme concentration were investigated. When 50 μl 50% (v/v) CA, 100 μl 0.33 mM MA or 150 μl 1 mM PA was added to the mixture containing 2 ml 4.5 μM CPO and 500 μl 0.38 mM thioanisole, the biggest activities of these modified CPOs were observed (data not shown). Under the conditions, the degree of modification was about 60%. It indicates that about 3/5 amino groups seem to be modified.

Steady-state kinetics was measured for sulfoxidation and phenol oxidation by native and modified CPOs at a fixed concentration of hydrogen peroxide at room temperature in aqueous buffer and DMF. The oxidations follow Michaelis-Menten kinetics and the apparent kinetic parameters were shown in Table 1 and 2. Compared with native CPO, modified CPOs showed similar apparent Michaelis constant (*K*_m_) values and greater *k*_cat _values, thus the catalytic efficiencies *k*_cat_/*K*_m _were increased upon these modifications in aqueous buffer (Table 1). The catalytic efficiencies (*k*_cat_/*K*_m_) of CA-CPO, MA-CPO and PA-CPO for sulfoxidation in aqueous buffer were increased by 26.2%, 22.6%, and 12.9%, respectively. The catalytic efficiencies of CA-CPO, MA-CPO and PA-CPO for phenol oxidation in aqueous buffer were enhanced by 53.4%, 32.2% and 7.5%, respectively.

However, there are different trends caused by the modifications in organic solvent. Modified CPOs showed lower *K*_m _values and higher catalytic efficiencies *k*_cat_/*K*_m _for both sulfoxidation and phenol oxidation in 15% DMF. It indicates that these modifications increased the substrate affinities, hence enhanced its catalytic efficiencies. For sulfoxidation in DMF, modified CPOs had lower catalytic constants *k*_cat _than native CPO. The biggest increase in the catalytic efficiency of MA-CPO for sulfoxidation was observed about 1.5-fold. Khajeh et al. reported that *k*_cat_/*K*_m _of CA-modified α-amylase from *Bacillus licheniformis *was 3-fold higher that of native enzyme [25]. Our previous papers reported that PA-modified horseradish peroxidase showed a higher catalytic efficiency for the oxidation of phenol [22-24], and that MA- and CA-modified horseradish peroxidase had higher catalytic efficiencies for the oxidation of dye [20]. However, papains modified by CA, MA and PA showed lower catalytic efficiencies than native papain [26].

As shown in Table 1 and 2, we can find that the catalytic efficiencies of native and modified CPOs for sulfoxidation were higher than that for phenol oxidation in both aqueous buffer and DMF. It indicates that thioanisole is more easily oxidised than phenol by CPO.

From Table 1 and 2, we can also find that no significant changes of the catalytic efficiencies for sulfoxidation in aqueous buffer and DMF were observed. However, the catalytic efficiencies for phenol oxidation in DMF were lower than that in aqueous buffer. It may result from the difference of the energy of substrate desolvation [31]. Thioanisole is more energetically favourable for desolvation in DMF than phenol because of its higher hydrophobicity, hence retaining the catalytic efficiencies for sulfoxidation in DMF closer to that in aqueous buffer.

The dissociation constants of native and modified CPOs were determined by the spectral titration approach and the data were given in Table 3. These modifications decreased the dissociation constant (*K*_d_) for both thioanisole and phenol. It indicates that modified CPOs showed greater the substrate binding efficiencies. As shown in Table 3, we can also find that the dissociation constants of both native and modified CPOs for thioanisole were lower that for phenol, indicating higher binding efficiencies for thioanisole. It may arise from the higher hydrophobicity of thioanisole, leading to thioanisole more accessible into the active centre of enzyme.

### Stability

**Figure 1:**
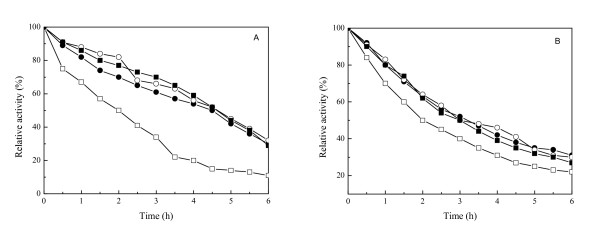
**Thermostability of native and modified CPOs at 50°C in aqueous buffer**. **A: peroxidase activity; B: Sulfoxidation activity**. CPO (■); CA-CPO (●); MA-CPO (□); PA-CPO (○). The values represent the mean of three independent sets of experiments with SD < 5%.

As indicated in Figure 1, treatments of CPO with these modification reagents resulted in a dramatic enhancement of its thermostability in aqueous buffer. After the exposure for 6 h at 50°C, native CPO retained only about 10% peroxidase activity and 20% sulfoxidation activity in aqueous buffer. Modified CPOs, however, retained about 30% peroxidase activity and 30% sulfoxidation activity. After single exponential fits of the thermal inactivation data, the apparent half-lives of native CPO, CA-CPO, MA-CPO and PA-CPO for peroxidase activity were 1.82 h, 3.94 h, 3.84 h and 3.93 h, respectively, k-values were 0.38, 0.18, 0.18 and 0.18, respectively. These values are only for stability comparison. The thermostability of peroxidase of CPO was increased about 2-fold upon these chemical modifications. The apparent half-lives of native CPO, CA-CPO, MA-CPO and PA-CPO for sulfoxidation activity were 2.71 h, 3.47 h, 3.10 h and 3.36 h, respectively. Thus, the thermostability of sulfoxidation activity of CPO was increased about 1.2-fold upon these chemical modifications. Chemical modification of horseradish peroxidase using CA, MA and PA brought about a dramatic enhancement of thermostability in aqueous buffer [21,24,27]. Modified papain by CA, MA and PA [26], and α-amylases by CA [25] also showed higher stability than native enzyme.

**Figure 2:**
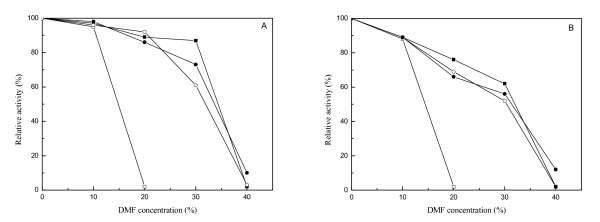
**Effects of solvents on native and modified CPOs at 30°C for 1 h**. **A: peroxidase activity; B: Sulfoxidation activity**. CPO (∀); CA-CPO (,); MA-CPO (!); PA-CPO (**○**). The values represent the mean of three independent sets of experiments with SD < 5%.

DMF tolerances of native and modified CPOs were compared. As shown in Figure 2, modified CPOs had a greater tolerance of DMF. After the exposure of native CPO in 20% DMF for 1 h at 30°C, the sulfoxidation and peroxidase activities were completely inactive. However, the exposure of modified CPOs in about 40% DMF for 1 h at 30°C just brought about the inactivity. Our previous papers also reported similar results for horseradish peroxidases modified by CA, MA and PA [21,22]. O'Brien et al. also reported that horseradish peroxidase modified by PA show higher thermostability and tolerance of DMF and THF [28].

The modification of ε-amino groups of the lysine residues (Figure 5) alters the positive charge to the negative charge (carboxylic group). CPO contains five lysine residues, Lys112, Lys115, Lys145, Lys177 and Lys211 [3]. The modification degrees of amino groups from CPO by CA, Ma and PA were determined as about 60% of the native enzyme. This result indicates that about 3/5 amino groups from native enzyme seem to be modified. Thus, the greater stabilities of modified CPOs likely arises from these modifications neutralizing positive lysine charges and then decreasing like-charge repulsions within the polypeptide as in the case of CA-, MA- and PA-modified horseradish peroxidase [21,22,24,28] and CA- and PA-modified papain [26]. Another possible explanation of modified CPOs' additional stabilities is the effect of the introduced hydrophilic groups. These introduced hydrophilic anchors may reduce the contact area of the exposed non-polar residues with the water in the unfolding state. In other words, the enhancement of surface hydrophilicity results in an increase in the essential energy for exposing hydrophobic groups to water in the unfolding process and therefore, prevents it from incorrect refolding [26,27]. A third possibility is that the bulky groups attached to the (modified) lysine residues provide new opportunity for the occurrence of the hydrogen bonding, hence prevent the unfolding and denaturation of the protein [26,27,32].

Immobilization of enzyme is still the most commonly used technique to improve the stability of enzyme. Sangeetha and Abraham demonstrated that immobilization of chemically modified enzyme is a useful strategy for reuse of enzyme as well as improving the thermal stability of the biocatalyst [26]. They reported that the stability of the modified papain was further increased by immobilization of the enzyme either by adsorption onto inert matrix or by entrapment in polysaccharide polymeric gels. Thus, the effects of immobilization of these modified CPOs on activity/stability would be further investigated.

### Spectra

**Figure 3:**
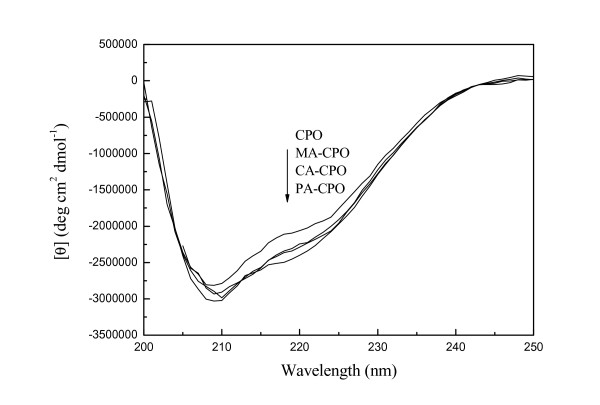
**CD spectra of native and modified CPOs in 10 mM acetate buffer (pH 5.0)**. Enzyme concentration was 7.5 μM.

In order to explore the mechanism of the enhancement of activities and stabilities upon these modifications, CD and fluorescence spectra of native and modified CPOs were determined. CD spectra in UV and UV-Vis regions provide information on the structure of protein and prosthetic heme of CPO. Fig. 3 shows CD of native and modified CPO in water. Native and modified CPOs have identical CD spectra with negative bands at 208 and 220 nm (Figure 3), which agreed with the previous results [16]. The spectral shapes of both modified CPOs are almost identical to those of native CPO. The percentages of secondary structure elements calculated using K2D software. No significant changes in the secondary structure content were observed by both modifications (37% α-helix, 26% β-structure and 38% random coil).

**Figure 4:**
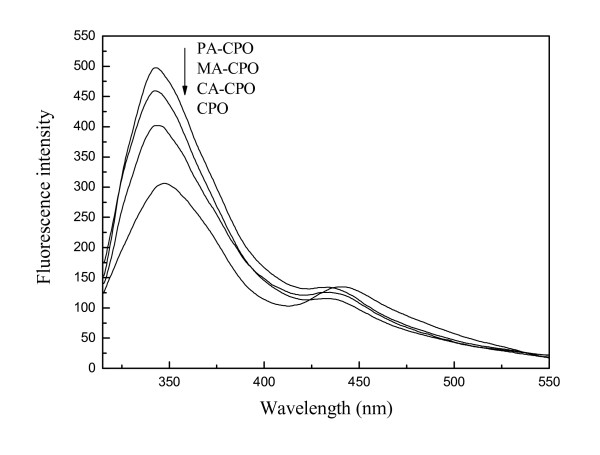
**Fluorescence spectra of native and modified CPOs in 10 mM acetate buffer**. Excitation wavelength was 295 nm; Enzyme concentration was 7.5 μM.

**Figure 5:**
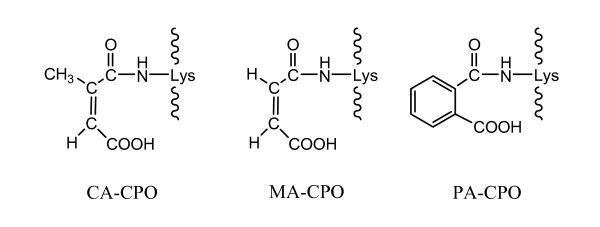
**Modification of ε-amino groups of lysines of CPO**.

The fluorescence emission excited at 295 nm arises solely from the tryptophan residues. Thus, the tryptophan fluorescence was used to probe the structural change of modified CPOs compared to native CPO. Figure 4 shows that the tryptophan fluorescence emission blue shifted from 347 nm of native CPO to 343 nm of modified CPOs. It indicates that the tryptophan residue of the modified CPOs became less exposed and located in a less polar environment. From Figure 4, we can also find that the intensities of the tryptophan fluorescence emission were increased after modifications because of a change in the relative orientation or distance between the heme and the tryptophan residue leading to a decrease in the efficiency of energy transfer [33]. This also denotes that the distance between the heme and the tryptophan residue increased [34]. Another explanation for PA-CPO having the biggest intensity of fluorescence may be PA modification introduced a bulky benzene ring structure onto the lysine side chain [22].

CD and fluorescence is a useful conformation probe for the studying of heme proteins. The changes of modified other heme-protein have been detected using spectroscopic technique. PEG-Met-hemoglobin showed up to 10-fold higher activity, greater substrate affinity and the catalytic efficiency (*k*_cat_/K_m_) than unmodified protein [35]. They also found that there was a red shift on the Soret band after modification. Carboxymethylated cytochrome *c *had greater catalytic activity and higher tryptophan fluorescence intensity than native cytochrome *c *[36]. PA-, MA- or CA-modified horseradish peroxidases showed similar catalytic and spectrum properties to these modified CPOs [21,22].

## Conclusion

Chemical modification of CPO by CA, MA or PA increased their catalytic efficiencies, and thermostabilities in both aqueous buffer and DMF. In aqueous buffer, modified CPOs showed a similar *K*_m _values and greater catalytic efficiencies *k*_cat_/*K*_m _for both sulfoxidation and phenol oxidation compared to native CPO. However, in organic solvent (DMF), modified CPOs had lower *K*_m _values and higher catalytic efficiencies *k*_cat_/*K*_m _than native CPO. These modifications also improved their thermostabilities by 1~2-fold and solvent tolerances of DMF. Fluorescence spectra proved that these modifications changed the environment of tryptophan. The improvements of the activity and stability are related to side chain reorientations of aromatics upon both modifications.

## Methods

### Reagent

Chloroperoxidase from *Caldariomyces fumago *was purchased from Fluka and had a specific activity of 11400 U/ml. Citraconic anhydride and thioanisole were purchased from Alfa Aesar. Phthalic anhydride and maleic anhydride were of analytic grade and obtained from Guangzhou Chemical Reagent Factory. All other reagents were of analytic grade.

### Chemical modification

Chemical modification was based on our previous method [20-24]. CA, MA, thioanisole and chloroperoxidase were dissolved in 50 mM sodium citrate buffer (pH 5.0) and PA was dissolved in 50 mM sodium citrate buffer (pH 5.0) containing 4% dimethylsulfoxide. 500 μl 0.38 mM thioanisole and 2 ml 4.5 μM CPO were first mixed at 4°C and then 50 μl 50% (v/v) citraconic anhydride, 100 μl 0.33 mM maleic anhydride or 150 μl 1 mM phthalic anhydride was added to the mixture. The reaction proceeded at 4°C for 1 h and was finally dialyzed against 50 mM sodium citrate buffer (pH 5.0) at 4°C to removal excess reagents.

The degree of modification was estimated by the trinitrobenzensulphonic acid method of Fields [37]. The concentration of CPO was determined from its Soret absorbance (molar extinction coefficient at 400 nm = 91200 M^-1^cm^-1^) [38].

### Activity assay

All UV-visible absorption measurements were determined in a Shimadzu UV2450 spectrophotometer using 1-cm path length quartz cuvettes.

#### Monoxygenase activity

The sulfoxidation of thioanisole was used to measure monooxygenase activity according to the method of Spreti et al. [30]. The reaction mixture contained 50 mM sodium citrate buffer (pH 5.0), 126 μM thioanisole, 300 μM H_2_O_2 _and 0.1 ml enzyme in a total volume of 3 ml. The enzymatic reaction was started by the addition of enzyme, and the activity was determined by monitoring the decrease in absorbance at 250 nm at room temperature (ε = 9700 M^-1^cm^-1^). One unit of sulfoxidation activity was defined by the amount (micromoles) of thioanisole consumed per minute under the assay conditions.

#### Peroxidase activity

The enzyme activity was assayed by colorimetric method [21]. Reaction mixture containing 10 mM phenol, 0.2 mM hydrogen peroxide and 2.4 mM 4-aminoantipyrin (4-AAP) in a total volume of 3.0 ml was incubated at 30°C. All reagents were dissolved in 50 mM sodium citrate buffer (pH 5.0). The reaction was then started by adding 0.1 ml of diluted enzyme solution, and the initial increase in absorbance was monitored at 510 nm during one minute. The rate of reaction is proportional to the enzyme activity and is deduced from the rate of formation of the nonprecipitating product which absorbs light at a peak wavelength of 510 nm with a molar extinction coefficient of 7100 M^-1^cm^-1^. One unit of activity (U) is defined as the number of micromoles of peroxide utilized per minute under the assay conditions.

#### Thermostability assay

Native and modified CPO preparations were incubated in 50 mM sodium citrate buffer (pH 5.0) at 50°C. Aliquots of each sample were withdrawn at different times and assayed for sulfoxidation and peroxidase activity under the standard conditions as stated above.

#### Catalytic stability in organic solvents

Organic solvent profiles of CPO samples were carried out at room temperature with exposure time of 1 h. The solvent used was dimethylformanmide (DMF). Reaction mixtures were comprised of 0.1 M sodium phosphate buffer (pH7.4) containing increasing 10% (v/v) increments of organic solvent. 100 μl were withdrawn from each reaction mixture and assayed under the standard conditions above.

#### Kinetics

The kinetic experiments for sulfoxidation of thioanisole were performed using constant enzyme and H_2_O_2 _concentration (300 μM) as the sulfoxidation activity assay, and varying the concentration of thioanisole under the same conditions of the activity assay.

The kinetic experiments for peroxidase were performed using constant enzyme, 4-AAP and H_2_O_2 _concentration (0.2 mM) as the peroxidase activity assay, and varying the concentration of phenol under the same conditions of the activity assay.

Data were fitted using non-linear least-squares program of Origin software (Version 7.0) to obtain kinetic parameters.

#### Substrate binding

Apparent dissociation constants of substrate (thioanisole or phenol) for CPO were determined by spectral titration on the Shimadzu UV2450 spectrophotometer at 25°C as described elsewhere [39]. A 260 μl solution of CPO (about 4.5 μM) in 50 mM sodium citrate buffer (pH 5.0) was added to the sample and reference cell, respectively. A few microlitters of concentrated substrate (378 μM thioanisole or 15 mM phenol) in 50 mM sodium citrate buffer (pH 5.0) were added to the CPO solution in the sample cell and an equivalent volume of buffer to the enzyme solution in the reference cell. Suitable amount of 50 mM sodium citrate buffer was added to the sample and reference cell to control the total volume of 0.8 ml. For titration studies, each solution was incubated at 25°C for at least 5 min to reach equilibrium after the substrate was added. And then the difference spectra of protein-substrate vs protein solutions were recorded. The obtained spectral changes caused by substrate addition were fitted to the following equation by a non-linear least-squares procedure:

ΔAsoret=ΔAs[S]Kd+[S]
 MathType@MTEF@5@5@+=feaafiart1ev1aaatCvAUfKttLearuWrP9MDH5MBPbIqV92AaeXatLxBI9gBaebbnrfifHhDYfgasaacH8akY=wiFfYdH8Gipec8Eeeu0xXdbba9frFj0=OqFfea0dXdd9vqai=hGuQ8kuc9pgc9s8qqaq=dirpe0xb9q8qiLsFr0=vr0=vr0dc8meaabaqaciaacaGaaeqabaqabeGadaaakeaacqqHuoarcqWGbbqqdaWgaaWcbaGaem4CamNaem4Ba8MaemOCaiNaemyzauMaemiDaqhabeaakiabg2da9maalaaabaGaeuiLdqKaemyqae0aaSbaaSqaaiabdohaZbqabaGccqGGBbWwcqWGtbWucqGGDbqxaeaacqWGlbWsdaWgaaWcbaGaemizaqgabeaakiabgUcaRiabcUfaBjabdofatjabc2faDbaaaaa@466C@

where Δ*A*_soret _and Δ*A*_*S *_are the absorbance changes of Soret band at a given and saturating substrate concentration respectively. [s] is the concentration of substrate.

### CD spectra

CD experiments were carried out using a Jasco J810 spectropolarimeter. CD was monitored with a cell of 2 mm path length with enzyme concentration of about 7.5 μM in 10 mM sodium acetate buffer (pH 5.0). CD spectra reported in the region were an average of two scans recorded at a scan speed of 250 nm/min, a slit width of 0.2 nm, a response time of 1 s and a resolution of 0.1 nm, corrected by subtracting the appropriate blank runs on CPO-free solutions. The CD data were expressed in terms of mean residue ellipticity, [θ], in deg cm^2 ^dmol^-1^. The secondary structure percentage predictions were made using K2D software [40].

### Fluorescence spectra

Fluorescence measurements were carried out using a LS 55 spectroflurimeter (PE). The intrinsic tryptophan fluorescence on excitation at 295 nm was recorded for emission from 300 nm to 550 nm. The slit widths for both the excitation and the emission monochromators were set at 10 nm, the scan speed at 540 nm/min and the resolution at 10 nm. The enzyme concentration was about 7.5 μM in 10 mM sodium acetate buffer (pH 5.0).

## Authors' contributions

JZL: developed the concept and designs of the method, guided the project, computed the secondary structure percentage and drafted the manuscript. MW: carried out most of experiments.

## Figures and Tables

**Table 1:** Kinetic parameters of native and modified CPOs in 50 mM citrate buffer (pH 5.0)

Enzyme	Sulfoxidation			Oxidation of phenol		
		
	*K*_m _(mM)	*k*_cat _(1000/min)	*k*_cat_/*K*_m _(1000/mM/min)	*K*_m _(mM)	*k*_cat _(1000/min)	*k*_cat_/*K*_m _(1000/mM/min)
CPO	1.64 ± 0.09	19.9 ± 0.6	12.1	0.35 ± 0.01	1.5 ± 0.1	4.3
CA-CPO	1.61 ± 0.08	24.6 ± 2.0	15.3	0.33 ± 0.02	2.1 ± 0.2	6.4
MA-CPO	1.45 ± 0.11	21.6 ± 0.9	14.9	0.32 ± 0.01	1.8 ± 0.1	5.6
PA-CPO	1.82 ± 0.16	24.9 ± 1.7	13.7	0.40 ± 0.03	1.8 ± 0.1	4.5

The values represent the mean of three independent sets of experiments with SD < 5%

**Table 2:** Kinetic parameters of native and modified CPOs in 15% DMF

Enzyme	Sulfoxidation			Oxidation of phenol		
		
	*K*_m _(mM)	*k*_cat _(1000/min)	*k*_cat_/*K*_m _(1000/mM/min)	*K*_m _(mM)	*k*_cat _(1000/min)	*k*_cat_/*K*_m _(1000/mM/min)
CPO	6.79 ± 0.54	75.4 ± 3.0	11.1	6.69 ± 0.26	6.4 ± 0.5	1.0
CA-CPO	1.43 ± 0.07	20.4 ± 0.8	14.3	5.30 ± 0.32	6.3 ± 0.4	1.2
MA-CPO	2.50 ± 0.22	41.4 ± 1.0	16.6	5.33 ± 0.48	6.4 ± 0.5	1.2
PA-CPO	2.31 ± 0.19	26.1 ± 1.6	11.3	4.87 ± 0.29	6.8 ± 0.4	1.4

The values represent the mean of three independent sets of experiments with SD < 5%

**Table 3:** Dissociation constants of native and modified CPOs

Substrate	*K*_d_							
	
	50 mM citrate buffer (pH 5.0)				15% DMF			
		
	CPO	CA-CPO	MA-CPO	PA-CPO	CPO	CA-CPO	MA-CPO	PA-CPO
thioanisole	0.56 ± 0.04	0.05 ± 0.03	0.11 ± 0.07	0.58 ± 0.06	1.05 ± 0.07	0.07 ± 0.04	0.35 ± 0.08	0.92 ± 0.04
phenol	21.04 ± 0.82	2.78 ± 0.46	8.51 ± 1.88	23.93 ± 2.88	122.70 ± 1.26	39.78 ± 2.58	13.70 ± 1.47	83.08 ± 3.96

The values represent the mean of three independent sets of experiments with SD < 5%
